# Genetic susceptibility to mercury in the amazon: A cross-sectional approach to snps analyses in exposed riverine populations

**DOI:** 10.1590/1678-4685-GMB-2025-0115

**Published:** 2026-08-14

**Authors:** Amanda Lopes-Araújo, Jean Ludger Barthelemy, Gabriela P. Arrifano, Letícia Santos-Sacramento, Isabela Soares-Silva, Caio G. Leal-Nazaré, Camila Lago-Pinheiro, Barbarella M. Macchi, Rosa C. Rodríguez Martín-Doimeadios, María Jiménez-Moreno, Marcus Augusto-Oliveira, Maria Elena Crespo-López

**Affiliations:** 1 Universidade Federal do Pará, Instituto de Ciências Biológicas, Laboratório de Fisisopatologia e Farmacologia Molecular, Belém, PA, Brazil.; 2 Instituto Amazônico do Mercúrio, Belém, PA, Brazil.; 3 Universidade Federal do Pará, Instituto de Ciências Biológicas, Laboratório de Neuroquímica Celular e Molecular, Belém, PA, Brazil.; 4 Universidad de Castilla-La Mancha, Facultad de Ciencias Ambientales y Bioquímica, Departamento de Química Analítica y Tecnología de los Alimentos, Toledo, España.

**Keywords:** Amazonian, methylmercury, polymorphism, human, gene-environment interaction

## Abstract

Mercury is among the worst global pollutants being a significant public health concern in the Amazon, primarily due to contaminated fish intake. The relationship between mercury body burden and genetic polymorphisms in *GSTP1* (rs1695), *PON1* (rs662), *MT1A* (rs11076161), and *SEPP1* (rs7579) genes was analyzed in Amazonian riverine populations, with a rapid and cost-effective approach by selecting individuals at both extremes of the exposure spectrum. Genotyping revealed significant associations for *GSTP1* and *PON1* polymorphisms: the G allele of *GSTP1* and the CC genotype of *PON1* were more frequent among highly exposed individuals, suggesting reduced detoxification efficiency. Additionally, although non-significant, a higher prevalence of the GG and CC genotypes were observed for *MT1A* and *SEPP1*, respectively, in the high-exposure group, pointing to the need for further investigation. These findings enhance our understanding of how genetic variation influences individual susceptibility to mercury accumulation in the Amazon and can support public health prevention strategies and the early identification of high-risk individuals within these vulnerable populations. Furthermore, this study underscores the importance of considering gene-environment interactions in environmental health assessments and highlights the need for public health strategies tailored to the genetic and socio-environmental contexts of vulnerable Amazonian communities.

## Introduction

The Amazon Basin is one of the most ecologically diverse and culturally rich regions in the world. Spanning across nine South American countries (Bolivia, Brazil, Colombia, Ecuador, Guyana, Peru, Suriname, Venezuela, and French Guiana) and housing the largest tropical rainforest on the planet, the Amazon is home to thousands of species of plants and animals, as well as hundreds of Indigenous and traditional communities whose livelihoods are closely intertwined with the natural environment (Galvis, 2019). Among traditional communities of the Amazon, riverine populations depend heavily on the rivers for transportation, water, and especially for food, because fish constitutes their primary source of animal protein, cultural identity, and economic sustenance (Machado et al., 2021).

The Amazon has also become a region of mounting environmental concern due to deforestation, hydroelectric development, and, notably, gold mining over the past several decades (Arrifano et al., 2018 a ; Crespo-Lopez et al., 2021, 2023a; Augusto-Oliveira et al., 2025). One of the most pernicious consequences of the latter activity is the widespread contamination of water bodies with mercury, particularly in its organic and most toxic form, methylmercury (MeHg). Mercury released into aquatic systems undergoes microbial methylation, and MeHg then accumulates in the aquatic food chain, reaching high concentrations in predatory fish species commonly consumed by local populations (Rodriguez Martin-Doimeadios et al., 2014; Basta et al., 2023; Augusto-Oliveira *et al*., 2025). As a result, many Amazonian communities experience chronic dietary exposure to MeHg at levels that far exceed those considered safe by international health agencies (Arrifano *et al*., 2018b; Santos-Sacramento et al, 2021; Lopes-Araújo et al., 2023; Augusto-Oliveira *et al*., 2025). 

Worryingly, mercury is considered one of the worst global pollutants especially affecting human health (Arrifano et al., 2023). The exposure poses serious risks, especially to the Central Nervous System (CNS), due to mercury’s ability to cross the blood-brain barrier, causing neurological deficits, motor impairments, and cognitive dysfunction (Crespo-Lopez et al., 2022 and 2023b). Despite the underreporting in official records, numerous studies have confirmed elevated concentrations of mercury in hair samples from individuals living in the Amazon, underscoring the public health crisis faced by these communities (Santos-Sacramento et al., 2021; Crespo-Lopez *et al*., 2024).

Also, even at similar environmental exposures, not all individuals accumulate mercury in the same way or experience the same health outcomes. Considerable inter-individual variability has been observed in terms of mercury body burden and susceptibility to its toxic effects. This variability is not fully explained by differences in diet, age, sex, or region, supporting that genetic factors play a significant role in determining the toxicodynamic and toxicokinetic properties of mercury for everyone (Llop et al., 2015; Andreoli and Sprovieri, 2017; Crespo-Lopez et al., 2023c). For instance, our previous findings demonstrated the influence of the apolipoprotein E gene in Amazonian populations exposed to mercury (Arrifano et al., 2018 c , 2018d), while no significant associations were identified for other apolipoproteins gene variants (Lopes-Araújo et al., 2023).

In the era of One Health (a collaborative, transdisciplinary approach that recognizes the interconnectedness of human and environmental health), environmental health genomics thus acquires essential importance. Recent advances in this area have identified several genetic polymorphisms that may influence mercury toxicokinetics and toxicity (Llop et al., 2015; Andreoli and Sprovieri, 2017; Crespo-Lopez et al., 2023 c ). Our recent review showed that single nucleotide polymorphisms (SNPs) with high worldwide frequency in genes such as *glutathione S-transferase Pi 1* (*GSTP1* for the gene - HGNC:4638, GSTP1 for the protein), *paraoxonase 1 (PON1* for the gene - HGNC:9204, PON1 for the protein*)*, *metallothionein 1A* (*MT1A* for the gene - HGNC:7405; MT1A for the protein), and *selenoprotein P* (*SEPP1* for the gene - HGNC:10760, SEPP1 for the protein), are involved in mercury transport, detoxification, and oxidative stress response, and have been associated with altered enzyme activity, expression levels, or cellular defense mechanisms (Crespo-Lopez *et al*., 2023c). However, no studies involving Amazonian populations were available for certain genes (such as *PON1*, *MT1A*, and *SEPP1*) or existing studies presented conflicting results, as in the case of the *GSTP1* (Crespo-Lopez *et al*., 2023c). Therefore, traditional populations of the Amazon, who are among the most exposed groups globally (Basu et al., 2018; Sharma et al., 2019), remain underrepresented in this body of research. Understanding how genetic variation affects these populations is not only a matter of scientific importance but also a question of environmental justice and health equity.

In this context, the present study sought to investigate the relationship between mercury exposure levels and genetic variations on *GSTP1*, *PON1*, *MT1A*, and *SEPP1*, in Amazonian riverine populations. A rapid and cost-effective approach was employed by initially measuring hair mercury concentrations in all participants, followed by the selection of 50 individuals matched by sex and age for subsequent genetic analysis. This approach allows for the integration of environmental monitoring with molecular epidemiology to advance our understanding of individual-level susceptibility to mercury toxicity in the Amazon. The findings have potential implications for refining risk assessments, informing public health policies, and identifying vulnerable subpopulations who may benefit from targeted interventions. Moreover, by focusing on traditional communities in the Global South, this study contributes to addressing the persistent geographic and socioeconomic inequities in environmental health research and response.

## Materials and Methods

### Study design and population

**Figure 1 -:**
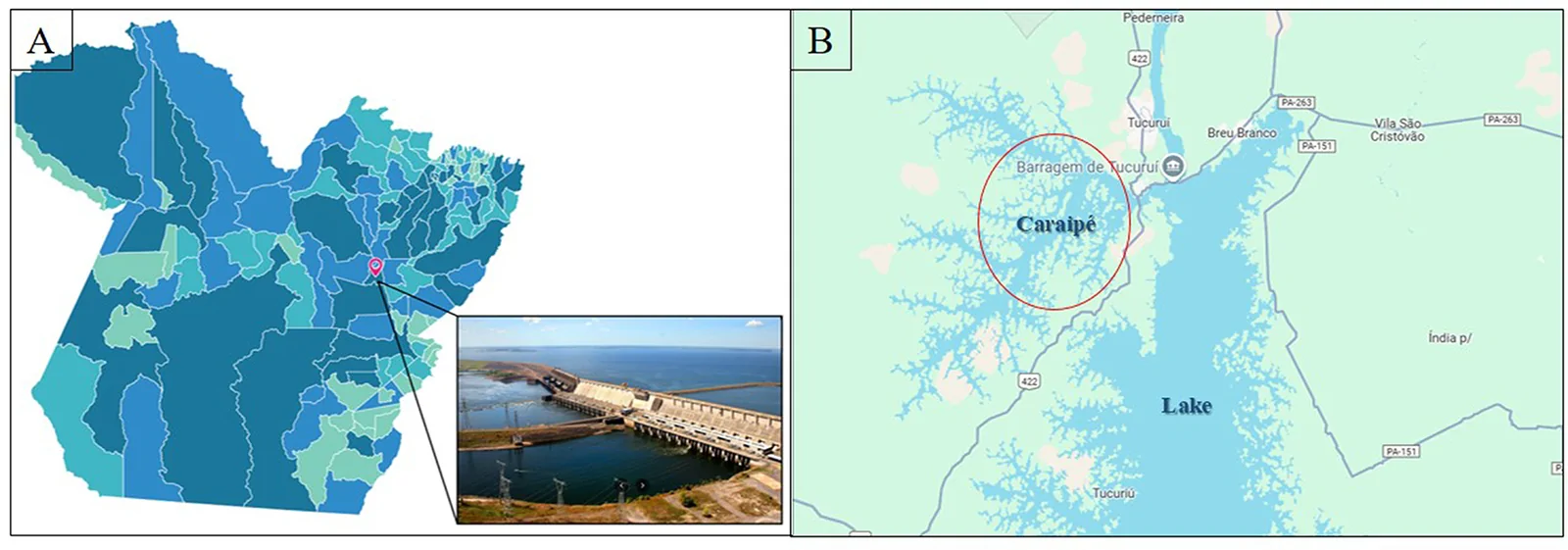
Tucuruí region. (A) Map of the state of Pará showing the municipality of Tucuruí and an image of the Tucuruí Hydroelectric Power Plant (adapted from https://cidades.ibge.gov.br/brasil/pa/tucurui/panorama). (B) Detailed map of the Tucuruí Lake area highlighting the Caraipé region, located near the dam (adapted from Google Maps).

Individuals from riverine communities of the Tucuruí Lake were included in the present study (Figure 1). The city of Tucuruí, located in the southeastern region of the state of Pará (latitude 03° 45’ 58’’ S and longitude 49° 40’ 21’’ W), is home to the Tucuruí Hydroelectric Power Plant (HPP), one of the largest dams in the world. According to the most recent data from the Brazilian Institute of Geography and Statistics (IBGE, 2022), the municipality covers a territorial area of 2,084.289 km² and has a population of approximately 91,306 inhabitants, including urban and rural populations. Despite the presence of the Tucuruí HPP, many of the traditional communities living in the islands of Tucuruí Lake still lack regular access to electricity and basic sanitation. These populations maintain dietary patterns that are heavily dependent on fish consumption (Crespo-Lopez et al., 2021; Machado et al., 2021; Lopes-Araújo et al., 2023). The communities included in this study are from the Caraipé region (latitude -3º 47’ 41” S, longitude -49º 48’ 3”), located on the riverine islands under HPP influence (Figure 1).

Participants were adults aged 18 to 70 years, of both sexes, who had lived in these regions for at least two years and reported consuming fish in five or more meals per week. According to hair mercury, 50 individuals were selected for SNPs analyses: 25 with low and 25 with high mercury exposure, matched by age. 

### Collection of anthropometric and demographic data

Data were collected during annual expeditions conducted between 2015 and 2018 in the Caraipé region of the Tucuruí Lake (Figure 1). The participants’ sex, age, weight, and height were recorded. Height was measured using a tape measure fixed vertically at the collection site, with the participant standing upright and barefoot. Body weight was measured using a calibrated digital scale. Body mass index (BMI) was calculated using the equation: weight (kg) divided by height squared (m^2^).

### Collection of biological samples

Venous blood samples (2 mL) were collected via venipuncture and stored in EDTA-coated BD Vacutainer tubes at -20ºC until analysis. Approximately 0.1g of hair was collected from the occipital region (1-2 cm from the scalp), using stainless steel scissor sanitized with ethanol (pure) before each collection. The samples were then stored in properly labeled paper envelopes.

### Extraction and quantification of total mercury and methylmercury

Mercury extraction and quantification were conducted according to Arrifano et al. (2018 a , 2018d). Briefly, mercury was extracted from approximately 0.1 g of hair by acid digestion with 10 mL of 6N nitric acid using a closed-vessel microwave digestion system (80°C for 5 minutes). The resulting clear extracts were analyzed for total mercury content by Inductively Coupled Plasma Mass Spectrometry (ICP-MS, ThermoFisher), after appropriate dilution. For mercury speciation, 2 mL of the acid digest was adjusted to pH 3.9 using 5 mL of 0.1 M acetate buffer and 30% ammonia. Then, 2 mL of hexane and 500 µL of sodium tetraethylborate (NaBEt₄) (3%, w/v) were added. The mixture was manually shaken for 5 minutes and centrifuged at 6,000 g for 5 minutes. The organic phase was transferred to glass vials and stored at -18°C until analysis by gas Chromatography-Pyrolysis-Atomic Fluorescence Spectrometry (GC-pyro-AFS). The certified human hair reference material (ERM-DB001, Sigma-Aldrich, Brazil) was used for quality control.

### Exposure group classification

Participants were categorized into two exposure groups based on hair mercury concentration: low exposure (≤ 2,300 ng/g) and high exposure (> 2,300 ng/g). This classification follows World Health Organization (WHO, 2021) recommendations, which set a maximum weekly intake of 100 µg of mercury for a 60-kg individual-corresponding to approximately 2,300 ng/g in hair (Crespo-Lopez et al., 2021; 2023b). Participants were matched by age for comparative analysis.

### DNA extraction and genotyping of polymorphisms

Genomic DNA was extracted from 200 µL of whole blood using the PureLink^®^ Genomic DNA Mini Kit (Invitrogen), following the manufacturer’s instructions. DNA quantification was performed using a Qubit^®^ 3.0 Fluorometer (Invitrogen/Life Technologies) with the Qubit^®^ dsDNA BR Assay Kit.

The sequence of the *MT1A* (rs11076161), *GSTP1* (rs1695), *SEPP1* (rs7579), and *PON1* (rs662) (Table 1) were amplified by real-time PCR using the TaqMan^®^ SNP Genotyping Assays method (Applied Biosystems^TM^) on an AriaMx Real-time PCR System (Agilent), to determine the SNPs. Each reaction consisted of 5 µL TaqMan^®^ Genotyping Master Mix, 0.5 µL TaqMan® SNP Genotyping Assay specific for each gene and 50 ng of genomic DNA in a final volume of 10 μL. All reactions were performed in duplicate, passive reference dye (Rox) for normalization was used and negative controls were included in each run. PCR cycling conditions consisted of an initial denaturation at 95 °C for 10 min, followed by 45 cycles of 95 °C for 15 s and 60 °C for 1 min. Genotyping was performed using AriaMx 1.8 software (Agilent Technologies, Santa Clara, CA, USA) based on fluorophore signal detection.

**Table 1 -:** Genetic variants analyzed according to HGNC and HGVS nomenclature.

Gene	HGNC ID	RefSeq transcript	rsID	HGVS (coding DNA)	HGVS (protein)	Functional region
*MT1A*	7405	NM_005946.4	rs11076161	c.-198A>G	-	Promoter / 5′ regulatory region
*SEPP1*	10760	NM_005410.4	rs7579	c.*251G>A	-	3′ untranslated region (3′UTR)
*GSTP1*	4638	NM_000852.3	rs1695	c.313A>G	p.Ile105Val	Coding region (missense variant)
*PON1*	9204	NM_000446.6	rs662	c.575A>G	p.Gln192Arg	Coding region (missense variant)

Note: Gene symbols are reported according to the HUGO Gene Nomenclature Committee (HGNC) and are italicized throughout. Variant nomenclature follows the recommendations of the Human Genome Variation Society (HGVS) using RefSeq transcripts (NM_). The prefix “c.” indicates coding DNA sequence positions. Variants located upstream of the translation start site are denoted with negative numbering (c.-), whereas variants in the 3′ untranslated region are indicated with an asterisk (c.*). Protein changes are described using the “p.” notation when applicable. The symbol “-” indicates that no amino acid change is defined for non-coding variants.

### Data analysis

Normality was assessed using the D’Agostino-Pearson test. For data following a Gaussian distribution, group comparisons were performed using the Student’s t-test; otherwise, the Mann-Whitney U test was applied. Hardy-Weinberg equilibrium (HWE) was evaluated using the Chi-squared (χ²) test. Allelic and genotypic frequencies between groups were compared using Fisher’s exact test and Chi-squared test, as appropriate. A significance level of p < 0.05 was adopted.

Given the exploratory nature of this study and the use of an extreme exposure design, no formal *a priori* power calculation was performed. However, with a total of 50 genotyped individuals (25 in each exposure group), the study was adequately powered to detect moderate to large differences in genotype or allele frequencies between groups, particularly for variants with known functional relevance. This sample size is expected to detect effect sizes corresponding to odds ratios of approximately 2.0 or greater, while smaller genetic effects may have remained undetected. The selection of individuals at both extremes of the mercury exposure distribution was intended to enhance contrast and statistical efficiency in this resource-limited context.

### Consideration of demographic and lifestyle variables

Demographic and lifestyle variables, including sex, age, body mass index (BMI), fish consumption frequency, and duration of residence in the study area, were collected for all participants and evaluated descriptively. For the genetic analyses, individuals were intentionally selected from both extremes of the mercury exposure distribution and matched by age, minimizing potential confounding by this key demographic factor at the design stage. Fish consumption frequency and duration of residence showed limited variability within the selected riverine subsample, reflecting long-term residence and a largely homogeneous dietary pattern characterized by frequent fish intake. Given the relatively small size of the genotyped subgroup (n = 50), additional multivariable adjustment was not performed in order to avoid model overfitting and unstable estimates. Therefore, genotype-exposure associations were assessed using a parsimonious analytical approach focusing on direct comparisons between exposure groups.

### Ethical issues

This work was according to the STROBE guideline (von Elm et al., 2007) and followed the ethical principles of the Declaration of Helsinki for human research. All procedures were approved by the Human Research Ethics Committee in Brazil (CAAE No. 43927115.4.0000.0018), and written informed consent was obtained from all participants.

## Results

The total study population comprised 414 individuals, 40.1% of whom were male and 59.9% female (Table 2). Regarding anthropometric measurements, the median body mass index (BMI) exceeded the ideal values established by the World Health Organization (WHO, 2021). To assess mercury exposure, total mercury (THg) concentrations and the proportion of methylmercury (MeHg%) were quantified in hair of all participants (Table 2). The median THg level in the total population was 7,991 ng/g (interquartile range: 3,711-14,990 ng/g), with, on average, more than 87.8% of mercury found in the form of MeHg; this was consistent with the primary route of exposure described for traditional communities of Tucuruí Lake: frequent consumption of contaminated fish (Machado et al., 2021). 

**Table 2 -:** Demographic characteristics of the riverine population of Tucuruí Lake (Caraipé region). Data presented as medians and interquartile ranges (non-parametric) for all participants.

		Tucuruí n= 414
Gender	Male	166 (40.1%)
	Female	248 (59.9%)
Age (years)		45 (33-56)
Height (m)		1.56 (1.51-1.63)
Weight (kg)		65.2 (57.2-75.7)
BMI (kg/m²)		26.2 (23.4-29.8)
THg (ng/g)		7,991 (3,711-14,990)
MeHg (%)		87.8 (84.4 - 91.0)

Note: BMI, body mass index; THg, total mercury in hair; MeHg, methylmercury.

To investigate a possible association between genotypic distributions and mercury body burden, participants were classified into two groups based on THg concentrations in hair, and 50 individuals matched by age were selected at both extremes of the exposure spectrum (Table 3). The highly exposed group had not only a significantly higher concentration of THg in their hair (median of 29,990 ng/g, compared to 1,279 ng/g in the low exposure group; p < 0.0001), but also a higher proportion of MeHg, the most toxic and bioaccumulative form of the metal (with 90.5% in the highly exposed group versus 77.2% in the low exposure group; p = 0.0005). These results reinforce the differentiated exposure profile between groups in the same community and the importance of investigating how genetic factors can modulate this body burden of mercury.

**Table 3 -:** Demographic characteristics and mercury content of groups with low and high concentrations of total mercury (THg) from Tucuruí Lake. Data presented as mean and standard deviation (parametric) and medians and interquartile ranges (non-parametric).

		Total n= 50	Low exposed n=25	High exposed n=25	P value
Gender	Male	17 (34%)	1 (4%)	16 (64%)	< 0.0001^a^
	Female	33 (66%)	24 (96%)	9 (36%)	
Age (year)		43 ± 12	42 ±(12	43 ± 12	0.9107^b^
Height (m)		1.58 ± 0.08	1.57 (1.52-1.61)	1.59 ± 0.09	0.1829^c^
Weight (kg)		61.7 (57.9-73.85)	62.2 ± 9.6	70.3 ± 18.3	0.0570^c^
BMI (kg/m²)		25.1 (23.0-28.7)	25.0 (22.5-26.9)	25.7 (23.7-30.9)	0.2452^c^
THg (ng/g)		19,570 ± 21.24	1,279 ± 0.57	29,990 (26,840-45,310)	< 0.0001^c^
MeHg (%)		89.65 (86.62-91.98)	77.18 ± 9.74	90.5 ± 3.03	0.0005^b^

Note: BMI, body mass index; THg, total mercury in hair. ^a^ Fisher’s exact test. ^b^ t-Test. ^c^ Mann Whitney test.

Table 4 shows the genotype and allele distribution of the polymorphisms investigated in the *MT1A* (rs11076161), *SEPP1* (rs7579), *GSTP1* (rs1695) and *PON1* (rs662), as defined in Table 1. All genotypes were in Hardy-Weinberg Equilibrium, indicating that the observed genotype frequencies did not differ from those expected for a population in genetic equilibrium. Regarding genotype distribution, the most frequent were AG for *MT1A*, CC for *SEPP1*, AG for *GSTP1* and CT for *PON1* (Table 4). Also, the comparison of distributions of genotypic and allelic frequencies of the four polymorphisms between individuals with lower and higher mercury (Hg) exposure in the Tucuruí population was analyzed (Table 5). All SNPs evaluated were in Hardy-Weinberg equilibrium in both groups. Genotypic analyses revealed statistically significant differences between the low- and high-exposure groups for the *GSTP1* (rs1695) and *PON1* (rs662) polymorphisms (p = 0.0218 and p = 0.0408, respectively). For *GSTP1*, a higher frequency of the AA genotype was observed in the low-exposure group (37%), while a higher proportion of the AG genotype (60%) was found among individuals with high exposure, suggesting a possible association between the presence of the G allele and increased susceptibility to mercury bioaccumulation. Regarding *PON1*, there was a higher prevalence of the TT genotype in the low-exposure group and of the CC genotype in the high-exposure group, indicating a potential modulatory role of this gene in the response to mercury exposure.

**Table 4 -:** Genotypic and allelic distributions of *MT1A, SEPP1, GSTP1* and *PON1* in the Tucuruí Lake riverine population.

Genotypes	Total	
	n	(%)
*MT1A* rs11076161		
A/A	4	8%
A/G	23	47%
G/G	22	45%
Total	50	100%
*HWE, p-value*	0.8370	
A	31	32%
G	67	68%
*SEPP1* rs7579		
C/C	28	56%
C/T	20	40%
T/T	2	4%
Total	50	100%
*HWE, p-value*	0.7923	
C	76	76%
T	24	24%
*GSTP1* rs1695		
A/A	14	29%
A/G	25	51%
G/G	10	20%
Total	49	100%
*HWE, p-value*	0.9820	
A	53	54%
G	45	46%
*PON1* rs662		
C/C	9	18%
C/T	22	44%
T/T	19	38%
Total	50	100%
*HWE, p-value*	0.8406	
C	40	40%
T	60	60%

Note: HWE: Hardy-Weinberg Equilibrium.

**Table 5 -:** Distribution of genotypic and allelic frequencies of polymorphisms, according to exposure levels.

SNP	Genotypes and Alleles	Low exposed (n=25)	High exposed (n=25)	P value
*GSTP1* rs1695	AA	9 (37%)	5 (20%)	**0.0218^a^**
	AG	10 (42%)	15 (60%)	
	GG	5 (21%)	5 (20%)	
	A	28 (58%)	25 (50%)	0.4255^b^
	G	20 (41%)	25 (50%)	
HWE		0.7828	0.6065	
*PON1* rs662	CC	4 (16%)	5 (20%)	**0.0408^a^**
	CT	11 (44%)	11 (44%)	
	TT	10 (40%)	9 (36%)	
	C	19 (38%)	21 (42%)	0.8384 ^b^
	T	31 (62%)	29 (58%)	
HWE		0.9467	0.8893	
*MT1A* rs11076161	AA	3 (12%)	1 (4%)	0.5473^a^
	AG	12 (48%)	11 (46%)	
	GG	10 (40%)	12 (50%)	
	A	18 (36%)	13 (27%)	0.3896 ^b^
	G	32 (64%)	35 (73%)	
HWE		0.9785	0.7343	
*SEPP1* rs7579	CC	12 (48%)	16 (64%)	0.5037^a^
	CT	12 (48%)	8 (32%)	
	TT	1 (4%)	1 (4%)	
	C	36 (72%)	40 (80%)	0.4829 ^b^
	T	14 (28%)	10 (20%)	
HWE		0.6354	1.0000	

Note: HWE, Hardy-Weinberg Equilibrium. ^a^ Fisher’s Exact Test. ^b^ Chi-square tests, respectively.

**Figure 2 -:**
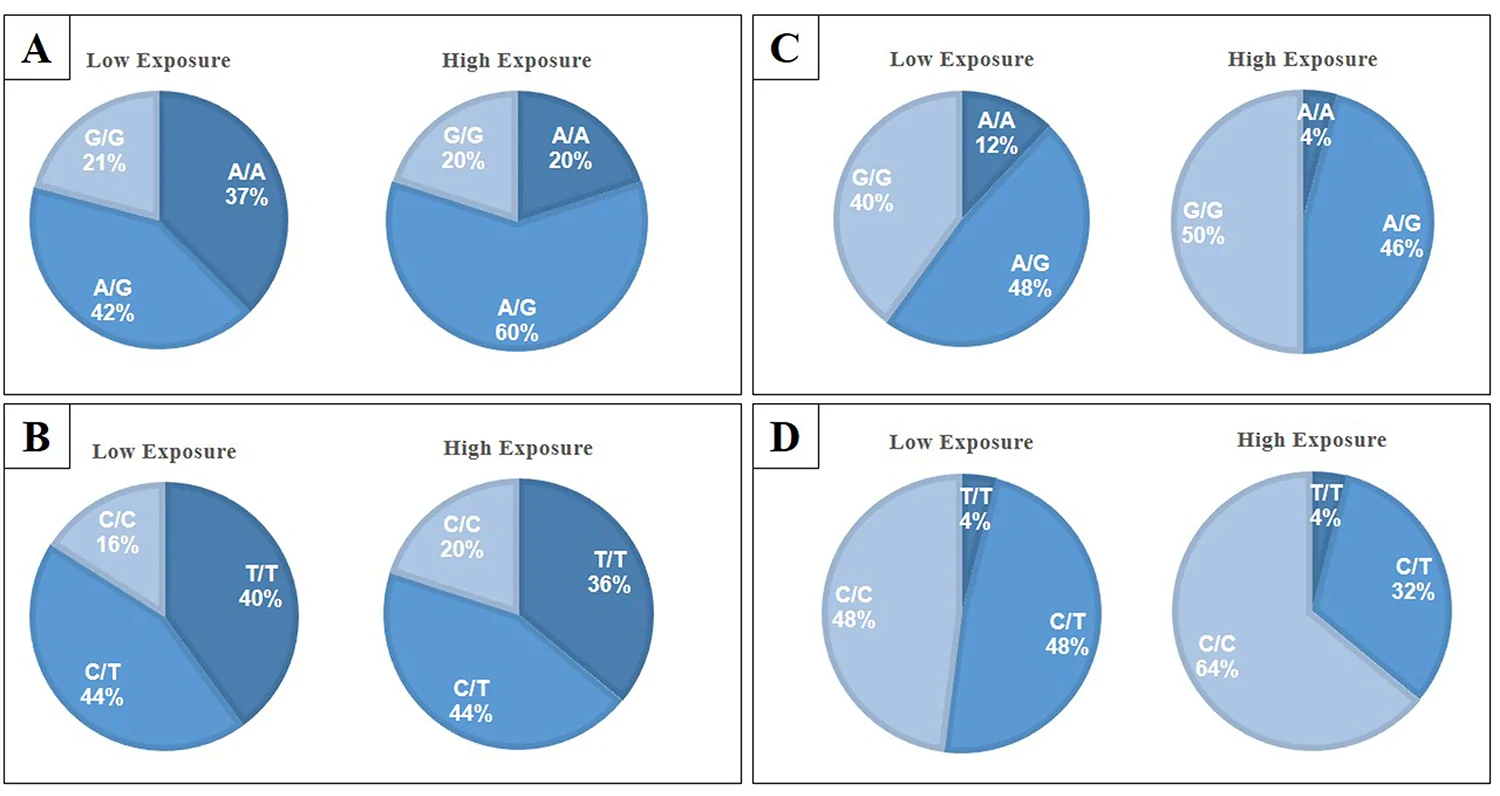
Genotypic distribution of GSTP1 (A), PON1 (B), MT1A (C), and SEPP1 (D) polymorphisms in mercury exposure groups of the riverine population from the Tucuruí Lake region.

On the other hand, the *MT1A* (rs11076161) and *SEPP1* (rs7579) polymorphisms showed no statistically significant differences in genotype frequencies between the exposure groups (Figure 2). However, there was a trend toward a higher frequency of the GG genotype for *MT1A* and a higher prevalence of the CC genotype for *SEPP1* among individuals with high exposure, suggesting a potential biological association that needs further investigation in studies with larger sample sizes.

## Discussion

The present study sought to explore the potential genetic susceptibility to mercury exposure in riverine population of the Brazilian Amazon (Tucuruí Lake), by analyzing polymorphisms in four genes (Table 1) that are functionally implicated in metal detoxification, oxidative stress response, and selenium transport. This population has been chronically exposed to mercury, primarily through the consumption of contaminated fish (Crespo-Lopez et al., 2021; Machado et al., 2021). By showing current mercury exposure in 414 people living in Tucuruí Lake as well as selecting and genotyping a set of individuals at both extremes of the exposure spectrum (50 participants with low and high mercury exposure), our findings provide new evidence of gene-environment interactions that may underlie differential vulnerability to mercury’s toxic effects. This rapid and cost-effective approach could be particularly useful in resource-limited settings such as much of the Amazon.

The overall mercury levels here described in hair were significantly high (Table 2), with a median THg concentration (7,991 ng/g) exceeding the hair reference level of 2,300 ng/g equivalent to the provisional tolerable weekly intake recommended by the World Health Organization (WHO, 2021), reinforcing concerns about chronic mercury exposure in these populations. This human exposure was even higher than that observed in other regions of the Amazon, such as the Tapajós River basin, which has historically been associated intense gold mining activity (Arrifano et al., 2018 d ; Crespo-Lopez et al., 2023 b ). Interestingly, no significant gold mining activity has been detected in the Tucuruí region, pointing to food intake as the main pathway of exposure (Arrifano *et al*., 2018a; Machado et al., 2021). The percentage of MeHg relative to THg was high (Table 1) and comparable to those found in other contaminated regions of the Amazon, suggesting a common source (likely piscivorous fish) (Crespo-Lopez *et al*., 2021; Augusto-Oliveira et al., 2025). This observation is aligned with known dietary patterns in Amazonian communities (Machado *et al*., 2021), where fish constitutes a major source of protein and is the principal vector for MeHg exposure. The widespread and sustained ingestion of contaminated fish may underlie the elevated hair mercury presence, even in the large cities of the Amazon (Basta et al., 2023).

For SNP analyses, a rapid and cost-effective approach was employed by selecting and comparing a subsample of 50 individuals at both extremes of the exposure spectrum (Table 3). This sample size is approximately 25% of the mean sample size (209 ± 37 individuals) found in studies analyzing mercury toxicity in Amazonian populations (Santos-Sacramento et al., 2021), and similar to other genetic studies carried out with vulnerable populations of the Amazon (Carvalho et al., 2024). The highly exposed group exhibited not only a median THg concentration approximately 23 times higher than that observed in the low-exposure group (29,990 ng/g vs. 1,279 ng/g), but also a significantly greater proportion of MeHg (90.5% vs. 77.2%) (Table 2). The elevated proportion of MeHg in the highly exposed group is particularly relevant from a toxicological perspective, as MeHg is the most lipophilic, toxic, and slowly eliminated form of mercury (Branco et al., 2021). These findings indicate that not only the total mercury burden but also its chemical form is critical, as it directly influences the risk of adverse health effects. This phenomenon was also observed in our previous study (Lopes-Araújo et al., 2023), suggesting that at lower THg concentrations, the body remains capable of metabolizing Hg into its inorganic form, whereas at higher THg concentrations, the Hg detoxification system becomes saturated. The chemical composition of accumulated Hg may also be influenced by genetic differences in mercury metabolism and excretion, supporting the investigation of modulating genetic variants, such as those analyzed in this study.

All genotypes (*MT1A* (rs11076161), *SEPP1* (rs7579), *GSTP1* (rs1695), and *PON1* (rs662)) were in HWE. Conformity to HWE indicates that, within this sample, there is no evidence of significant evolutionary forces acting on these alleles. It also suggests that the sample was representatively collected and that systematic genotyping errors are unlikely, reinforcing the validity of the genetic data obtained with this approach. Moreover, when compared to allele frequencies reported in international genomic databases such as NCBI’s dbSNP (Sherry et al., 2001) and the 1000 Genomes Project (1000 Genomes Project Consortium *et al*., 2015), the allele distribution patterns observed in our study are similar to those described for Latin American populations. Importantly, the analysis of genotypic distributions in populations historically underrepresented in large-scale genomic projects (such as Amazonian riverine communities) enhances our understanding of global genetic diversity and contributes to identifying potential population-specific variations in susceptibility to heavy metal toxicity.

To evaluate whether specific genetic variants may confer differential susceptibility to mercury accumulation, we conducted genotyping for four polymorphisms (Table 1) with prior evidence of relevance to metal metabolism (reviewed by Crespo-Lopez et al., 2023c). Our results revealed significant associations for *GSTP1* and *PON1*, but not for *MT1A* or *SEPP1*, in relation to mercury exposure levels (Figure 2, Tables 4 and 5).

A recent study demonstrated that the *GSTP1* rs1695 polymorphism exhibits different allele frequencies with a predicted moderate impact in both Amazonian Indigenous and American populations compared to other populations, particularly East Asian populations (Carvalho et al., 2024). In our study, the higher frequency of the AG genotype among highly exposed individuals, in contrast to the predominance of the AA genotype in the low-exposure group, suggests a possible association of the G allele with increased retention or absorption of mercury (Table 5). This association is biologically plausible given the role of GSTP1 in metal conjugation and detoxification. The rs1695 polymorphism is located within the enzyme’s active site, where an amino acid substitution alters enzymatic activity (Goodrich and Basu, 2012). GSTP1 is a phase II detoxification enzyme involved in conjugating reduced glutathione to a wide variety of electrophilic compounds, including reactive mercury species. The rs1695 polymorphism results in an amino acid substitution (Ile105Val), which has been shown to reduce enzyme activity and thermal stability. Although this polymorphism has been studied in Amazonian populations (Klautau-Guimarães et al., 2005; Barcelos et al., 2013, 2015; Silva et al., 2023, 2024) and in other populations worldwide, conflicting results on the effect of rs1695 on mercury body burden remains has been reported across different studies. Some studies have reported that the A allele is associated with increased hair mercury in Indigenous Amazonians (Silva *et al*., 2023, 2024), in the erythrocytes of individuals from northern Sweden (Engström et al., 2008), and in the hair of dental professionals in Michigan (Goodrich *et al*., 2011). In contrast, the G allele has been associated with higher mercury hair of women in Hong Kong (Chan et al., 2020).

This association is further supported by an *in vitro* study investigating the impact of *GSTP1* rs1695 variants on enzyme activity and substrate affinity (Goodrich & Basu, 2012). The results demonstrated that allozymes containing the Ile105 variant (corresponding to the A allele of rs1695) exhibited greater catalytic efficiency and substrate affinity compared to those containing the Val105 variant (G allele). Additionally, the G allele variant was found to be more sensitive to inhibition by both inorganic mercury (HgCl₂) and methylmercury (MeHg), suggesting that this variant may impair the enzyme’s detoxification capacity. The conflicting results across studies regarding the effect of genetic polymorphisms on mercury body burden may be attributed to several factors, including differences in mercury exposure levels among study populations (Chan et al., 2020), the use of different biological matrices (hair, urine, blood), and interactions with environmental variables such as diet, nutritional status, and co-exposure to other contaminants.

Although some data already exist for the *GSTP1* rs1695 polymorphism in vulnerable Amazonian populations (Indigenous groups), this is the first time that *PON1* has been analyzed in populations from the Amazon. Our data on the *PON1* rs662 polymorphism indicate that the CC genotype, which was more frequent in the highly exposed group, may be associated with reduced mercury detoxification capacity, whereas the TT genotype, more common among the less exposed individuals, could suggest greater protection (Table 5). PON1 is an enzyme involved in the metabolism of organophosphates and the defense against oxidative stress, and its activity can be modulated by polymorphisms that affect enzyme stability and efficiency (Costa et al., 2017). In the context of mercury exposure, whose toxicity is associated with the induction of reactive oxygen species, less active PON1 variants may compromise the antioxidant response and facilitate mercury retention in the body. Thus, *PON1* polymorphisms can influence serum enzyme concentrations and, consequently, reduce its protective function in humans. Because PON1 activity is highly dependent on calcium, cations such as heavy metals may bind to the enzyme and inhibit its function (Joneidi et al., 2019). Among the various SNPs identified in *PON1*, rs662 is one of the five most common and extensively studied (Ashiq & Ashiq, 2021). This polymorphism influences catalytic activity and is involved in the hydrolysis of multiple substrates, as well as the oxidation of low-density lipoproteins (LDL) (Soflaei et al., 2023). Although previous studies have not consistently demonstrated a direct relationship between *PON1* genotypes and mercury detoxification (Ayotte et al., 2011; Austin et al., 2014), the C allele has been discussed as being potentially more sensitive to inhibition by metals (reviewed by Joneidi *et al*., 2019).

Despite the relatively low sample size for a genetic analysis, trends toward a higher prevalence of specific genotypes (GG for *MT1A* and CC for *SEPP1*) were observed in our study among individuals with higher mercury body burden (Figure 2, Tables 4 and 5). While these trends did not reach statistical significance in our sample, they suggest potential biological associations that warrant further investigation in studies with larger sample sizes. Their influence on the toxicodynamics of mercury remains largely unexplored in Amazonian populations. 

*MT1A* encodes a low-molecular-weight protein involved in metal homeostasis and cellular protection against oxidative stress induced by toxic metals such as mercury (Andreoli & Sprovieri, 2017). Variations in this gene can affect its ability to bind and detoxify metals, due to transcriptional alterations and changes in protein structure (Andreoli & Sprovieri, 2017). The apparently higher frequency of the GG genotype in the highly exposed group (Table 5) may indicate greater biological susceptibility to mercury bioaccumulation, a hypothesis that merits testing in future gene expression studies.

SEPP1 plays a central role in the systemic transport of selenium, an essential element involved in antioxidant defense and protection against metal toxicity (Broberg et al., 2015). It also acts to neutralize mercury-induced oxidative stress, and its reduced expression can compromise cellular responses to mercury intoxication (Chen et al., 2006; Woods et al., 2014). The T allele of *SEPP1* rs10760 is associated with lower mercury concentrations in urine and hair (Goodrich et al., 2011). These findings align with our observations, where an apparently higher frequency of the CC genotype among individuals with a high mercury burden raises the hypothesis that this variant may be associated with a less efficient antioxidant response, favoring mercury accumulation and its deleterious effects. 

However, the present data suggest that, if *MT1A* and *SEPP1* effects exist in this population, they are likely of smaller magnitude than those observed for *GSTP1* and *PON1* and may require larger sample sizes and multivariable approaches to be reliably detected. Accordingly, these observations should be viewed as hypothesis-generating and warrant further investigation in future studies.

### Strengths and limitations

Some limitations of this study should be acknowledged. The relatively small number of genotyped individuals may have reduced statistical power to detect small genetic effects. Nevertheless, by selecting individuals at both extremes of the mercury exposure spectrum, the study was optimized to detect moderate to large differences in genotype distribution between exposure groups. With 25 individuals per group, the design was sufficiently powered to identify associations of moderate magnitude, as observed for *GSTP1* and *PON1*, whereas weaker associations (such as the non-significant trends observed for *MT1A* and *SEPP1*) may require larger samples to reach statistical significance. Importantly, the absence of statistical significance for these variants should not be interpreted as evidence of absence of a biological effect.

Although demographic and lifestyle variables such as sex, BMI, fish consumption frequency, and duration of residence were collected, they were not included as covariates in multivariable genetic models. This decision was primarily driven by both the study design and sample size considerations. Individuals included in the genotyping analyses were matched by age and sex, reducing potential confounding by these variables *a priori*. Moreover, fish consumption frequency and duration of residence were largely homogeneous within the riverine communities studied, reflecting long-term residence and sustained reliance on fish as a dietary staple, and thus contributed limited variability within the selected subsample.

The relatively small number of genotyped individuals also constrained the inclusion of multiple covariates without risking overfitting and loss of statistical stability. Accordingly, a parsimonious analytical strategy was adopted, focusing on the identification of moderate to large genotype-exposure associations using an extreme exposure design. Future studies with larger sample sizes and greater heterogeneity in lifestyle and environmental factors will be essential to explore multivariable and interaction models and to further disentangle the combined influence of genetic, environmental, and behavioral determinants of mercury body burden. In addition, the inclusion of phenotypic biomarkers of effect, such as neurobehavioral outcomes or oxidative stress markers, would help to contextualize the functional implications of genetic susceptibility.

Despite these limitations, this study provides important contributions to the field of environmental health genomics in underrepresented populations. By focusing on Amazonian riverine communities, we address a significant research gap, as most gene-environment interaction studies have been conducted in high-income countries or occupationally exposed groups. Our approach, pairing participants by age, and selecting individuals at both extremes of the exposure spectrum, helped to isolate the effect of genetic variants under relatively homogeneous environmental conditions. Additionally, we did not assess phenotypic biomarkers of effect (e.g., neurobehavioral outcomes, oxidative stress markers), which would help to contextualize the functional implications of the observed genotypic differences. Future research should incorporate a systems biology approach, integrating genomics, transcriptomics, and metabolomics with detailed exposure and health outcome data.

Overall, our data highlights a concerning scenario of chronic mercury exposure in riverine populations of the Amazon, particularly in Tucuruí, and reinforces the hypothesis that genetic factors (such as polymorphisms in *GSTP1* and *PON1*) may contribute to individual variability in mercury body burden, even under similar environmental exposure conditions. These findings underscore the importance of investigating gene-environment interactions in traditionally underrepresented populations, such as those in the Amazon region, and emphasize the need for interdisciplinary approaches and public health strategies that are sensitive to the genetic and socio-environmental specificities of these communities. Identifying genetic variants associated with increased susceptibility to mercury toxicity can inform more targeted environmental surveillance efforts and support the development of public policies that recognize the heterogeneity of exposed populations. Furthermore, this research highlights the importance of environmental justice. Indigenous and traditional communities in the Amazon face disproportionate exposures to hazardous substances due to socio-political marginalization and lack of regulatory enforcement. Recognizing genetic susceptibility within these populations strengthens the ethical imperative for intervention and highlights the need for culturally appropriate, community-engaged public health programs in the Amazon.

## Data Availability

 The data that support the findings of this study are available on request from the corresponding authors.
